# Malnutrition among Patients with Chronic Liver Disease Admitted to the Department of Gastroenterology and Hepatology in a Tertiary Care Centre: A Descriptive Cross-sectional Study

**DOI:** 10.31729/jnma.8210

**Published:** 2023-07-30

**Authors:** Sandeep Raj Bajracharya, Denis Peeyush, Vivek Malla, Mrigendra Singh, Puspendra Tiwari, Sunil Gurung, Bickram Pradhan

**Affiliations:** 1 Department of Gastroenterology and Hepatology, B.P. Koirala Institute of Health Sciences, Dharan, Sunsari, Nepal

**Keywords:** *liver diseases*, *prevalence*, *protein-calorie malnutrition*

## Abstract

**Introduction:**

Protein-calorie malnutrition is a transversal condition to all stages of chronic liver disease. It is considered an independent predictor of mortality that leads to an increased number of complications. The aim of this study was to find out the prevalence of malnutrition among patients with chronic liver disease admitted to the Department of Gastroenterology and Hepatology in a tertiary care centre.

**Method:**

This descriptive cross-sectional study was done among patients with chronic liver disease admitted to the Department of Gastroenterology and Hepatology in a tertiary care centre between 10 January 2021 to 15 February 2022 after receiving ethical approval from the Institutional Review Committee (Reference number: 394/077/078-IRC). The nutritional status of patients was assessed by subjective global assessment. Convenience sampling was done. Point estimate and 95% Confidence Interval were calculated.

**Results:**

Among 110 patients with chronic liver disease, malnutrition was seen in 91 (82.72%) (75.65-89.79, 95% Confidence Interval). Out of 91 patients who were malnourished, 56 (61.53%) had moderate malnutrition whereas 35 (38.46%) had severe malnutrition. Seventy-six (83.51%) suffered from alcoholic cirrhosis.

**Conclusions:**

The prevalence of malnutrition among patients with chronic liver disease was higher than in other studies done in similar settings.

## INTRODUCTION

Protein-calorie malnutrition (PCM) is a common complication of chronic liver disease (CLD) and can have an adverse prognosis if left untreated. PCM is a condition involving cachexia due to nutritional deficiencies in calories and protein.^1^ Of those affected by CLD, approximately 20% with compensated cirrhosis and 65%-95% with decompensated cirrhosis have malnutrition.^2^ Malnutrition develops at an early stage of liver disease and its severity increases with the severity of liver disease.^3^

Nutritional assessment in CLD patients can be challenging. Hence, timely diagnosis of malnutrition should be done in such patients to improve the prognosis and quality of life.^4^

The aim of this study was to find out the prevalence of malnutrition among patients with chronic liver disease admitted to the Department of Gastroenterology and Hepatology in a tertiary care centre.

## METHODS

This descriptive cross-sectional study was done among patients of CLD admitted at the Department of Gastroenterology and Hepatology in B.P. Koirala Institute of Health Sciences, Dharan, Sunsari, Nepal after obtaining ethical approval from the Institutional Review Committee (Reference number: 394/077/078-IRC) where data collection was done between 10 January 2021 to 15 February 2022. All CLD patients who were admitted to the Department of Gastroenterology and Hepatology with an age >18 years, giving consent were included in the study. Patients with coexisting tuberculosis, HIV, hepatocellular carcinoma, and other malignancies and/or treatment with chemotherapy were excluded from the study. Convenience sampling was done. The sample size was calculated by using the following formula:


$$\begin{array}{ll} \text{n} & ={\text{Z}}^{2}\times \frac{\text{p}\times \text{q}}{{\text{e}}^{\text{2}}}\\ & =1.96^{2}\times \frac{0.76\times 0.24}{0.08^{2}}\\ & =110 \end{array}$$

Where,

n = minimum required sample size
Z = 1.96 at 95% Confidence Interval (CI)
p = prevalence of malnutrition taken from the previous study, 76.66%^5^
q = 1-p
e = margin of error, 8%

The calculated minimum required sample size was 110 and we took 110 patients.

Data was collected on a predetermined proforma covering the relevant subjects of the study. Detailed socio-demographic data was collected and the information was recorded in structured proforma. Chronic liver disease was diagnosed based on clinical, biochemical, endoscopic and radiological evidence of CLD. Informed consent was signed and confidentiality of the information was ensured.

Each patient was subjected to nutritional assessment using subjective global assessment. Using subjective global assessment (SGA), recent changes in weight, dietary intake, gastrointestinal symptoms, changes in functional capacity, perception of subcutaneous fat and muscle wasting, oedema and ascites were taken into account.^6^ Patients were classified as being well nourished (A), moderately malnourished (B) or severely malnourished (C). The severity of liver disease was defined using child-turcotte pugh (CTP) score based on clinical (ascites, encephalopathy) and laboratory parameters (albumin, bilirubin, prothrombin time, international normalised ratio (INR).^7^

Data were collected and entered in Microsoft Excel 2007 and analyzed in IBM SPSS Statistics version 16.0. Point estimate and 95% CI were calculated.

## RESULTS

Among 110 patients with chronic liver disease, 91 (82.72%) (75.65-89.79, 95% CI) were found to have malnutrition. Out of 91 patients who were malnourished, 56 (61.53%) had moderate malnutrition whereas 35 (38.46%) had severe malnutrition (Table 1).

**Table 1:** Nutritional assessment of CLD patients (n = 91).

Parameters	n (%)
Moderate malnutrition	56 (61.53)
Severe malnutrition	35 (38.46)

The mean age of patients was 50.00±12.84 years. Among 91 patients, 57 (62.63%) were male (Table 2).

**Table 2:** Baseline characteristics of participants (n = 91).

Characteristics	Categories	n (%)
**Gender**	Male	57 (62.63)
	Female	34 (37.36)

Alcohol-related liver disease was found in 76 (83.51%) patients. This was followed by chronic hepatitis C 4 (4.39%), chronic hepatitis B 3 (3.29%), NASH 2 (2.19%), Wilson's disease 2 (2.19%), cryptogenic cirrhosis 2 (2.19%), autoimmune hepatitis 1 (1.09%) and cholestasis 1 (1.09%) (Table 3).

**Table 3:** Aetiology of CLD (n = 91).

Categories	n (%)
Alcoholic liver disease	76 (83.51)
Chronic hepatitis C	4 (4.39)
Chronic hepatitis B	3 (3.29)
NASH	2 (2.19)
Autoimmune hepatitis	1 (1.09)
Cholestasis	1 (1.09)
Wilson's disease	2 (2.19)
Cryptogenic	2 (2.19)

**Figure 1:**
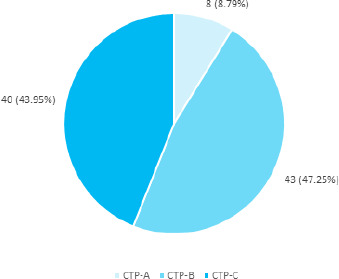
Classification according to CTP (n= 91).

Eight (8.79%) patients were CTP-A, 43 (47.25%) patients were CTP-B and 40 (43.95%) patients were CTP-C (Figure 1).

## DISCUSSION

The prevalence of malnutrition was found to be 91 (82.72%) among 110 patients with chronic liver disease. This finding was higher than in a similar study done in Nepal.^5^

The mean age of patients enrolled was 50.00±12.84 years, with the predominant patients being male and alcohol-related liver disease was the most common cause of CLD which was similar to the study done in Nepal^8^, in Africa^9^ and in the West.^10^ Most of the patients in this part of the world consume locally made alcoholic beverages. A study done in Nepal^11^ studied that drinking patterns and types of alcohol used showed a significant increase in the risk of developing CLD.

Nutritional assessment performed using SGA showed 82.72% were malnourished. These results showed that patients assessed in a ward setting presented more frequent and severe malnutrition. This fact may be explained by hypercatabolism, low food intake and loss of fat-free mass, which are exacerbated in decompensated disease. This study was comparable to studies done in Nepal^5^ and in the West.^12^

In the present study, PCM identified by SGA was associated with the severity of liver disease. Patients with more deteriorated functional status were especially CTB-B and CTP-C. The present study was comparable to a study done in Nepal^5^ and in west^12^ which showed similar results.

The limitation of this study was, this study included only hospitalized patients with CLD with acute decompensation who are known to be at an increased risk of malnutrition. Hence, the true prevalence of malnutrition in our context may have been slightly overestimated. The majority of our patients had alcoholic cirrhosis which may not be true in other populations.

In our study, anthropometry, dynamometry, bioimpedance and indirect calorimetry were not used to assess functional status, as the necessary equipment was not available during the period of the study. Also, since this is a single-centre study with a small sample size, the findings cannot be generalised to the whole country.

## CONCLUSIONS

The prevalence of malnutrition among patients with chronic liver disease was higher than in other studies done in similar settings. Hence, in CLD, nutritional assessment should be an integral part of patient evaluation.

## References

[1] Kalaitzakis E, Simren M, Olsson R, Henfridsson P, Hugosson I, Bengtsson M (2006). Gastrointestinal symptoms in patients with liver cirrhosis: associations with nutritional status and health-related quality of life.. Scand J Gastroenterol..

[2] Cheung K, Lee SS, Raman M (2012). Prevalence and mechanisms of malnutrition in patients with advanced liver disease, and nutrition management strategies.. Clin Gastroenterol Hepatol..

[3] Silva M, Gomes S, Peixoto A, Torres-Ramalho P, Cardoso H, Azevedo R (2015). Nutrition in chronic liver disease.. GE Port J Gastroenterol..

[4] Hasse J, Strong S, Gorman MA, Liepa G (1993). Subjective global assessment: an alternative nutrition-assessment technique for liver-transplant candidates.. Nutrition..

[5] Khadka D, Karki B, Thapa S, Khanal A, Shrestha R, Bhandary S (2019). Prevalence of malnutrition in patients with liver cirrhosis in a tertiary care hospital.. JNMA J Nepal Med Assoc..

[6] Detsky AS, Laughlin JR, Baker JP, Johnston N, Whittaker S, Mendelson RA (1987). What is subjective global assessment of nutritional status?. JPEN J Parenter Enteral Nutr..

[7] Child CG (1964). The liver and portal hypertension [Interent]..

[8] Sherpa TW, Pathak R, Khadga PK, Sharma S, Hamal R, Jha A (2019). Nutritional assessment of patients with liver cirrhosis by nutrition screening tool and anthropometry at a tertiary care center.. Journal of Institute of Medicine Nepal..

[9] Saleh MA, Mustafa HM, Eid KA, Soliman MM, Sultan HH (2014). Assessment of nutritional status of patients with chronic liver diseases admitted to gastroenterology department Al-Azhar University Hospital, Assiut. 2011.. AAMJ.

[10] Nunes G, Santos CA, Barosa R, Fonseca C, Barata AT, Fonseca J (2017). Outcome and nutritional assessment of chronic liver disease patients using anthropometry and subjective global assessment.. Arq Gastroenterol..

[11] Pradhan B, Hadengue A, Chappuis F, Chaudhary S, Baral D, Gache P (2015). Alcoholic liver disease in Nepal: identifying homemade alcohol as a culprit.. Clin Exp Gastroenterol..

[12] Vieira PM, De-Souza DA, Oliveira LC (2013). Nutritional assessment in hepatic cirrhosis; clinical, anthropometric, biochemical and hematological parameters.. Nutr Hosp..

